# Parental Burnout Assessment (PBA) in Different Hispanic Countries: An Exploratory Structural Equation Modeling Approach

**DOI:** 10.3389/fpsyg.2022.827014

**Published:** 2022-04-07

**Authors:** Denisse Manrique-Millones, Georgy M. Vasin, Sergio Dominguez-Lara, Rosa Millones-Rivalles, Ricardo T. Ricci, Milagros Abregu Rey, María Josefina Escobar, Daniela Oyarce, Pablo Pérez-Díaz, María Pía Santelices, Claudia Pineda-Marín, Javier Tapia, Mariana Artavia, Maday Valdés Pacheco, María Isabel Miranda, Raquel Sánchez Rodríguez, Clara Isabel Morgades-Bamba, Ainize Peña-Sarrionandia, Fernando Salinas-Quiroz, Paola Silva Cabrera, Moïra Mikolajczak, Isabelle Roskam

**Affiliations:** ^1^Carrera de Psicología, Facultad de Ciencias de la Salud, Universidad Científica del Sur, Lima, Peru; ^2^Centre for Multidisciplinary Research in Education, Russian Academy of Education, Moscow, Russia; ^3^Escuela de Psicología, Instituto de Investigación de Psicología, Universidad de San Martín de Porres, Lima, Peru; ^4^Facultad de Educación, Universidad Marcelino Champagnat, Lima, Peru; ^5^Facultad de Medicina, Universidad Nacional de Tucumán, San Miguel de Tucumán, Argentina; ^6^Center for Social and Cognitive Neuroscience, School of Psychology, Universidad Adolfo Ibáñez, Santiago, Chile; ^7^Carrera de Psicología, Facultad de Ciencias Sociales y Comunicación, Universidad Santo Tomás, Talca, Chile; ^8^Instituto de Psicología, Universidad Austral de Chile, Puerto Montt, Chile; ^9^School of Psychology, Millenium Institute for Research in Depression and Personality, Pontificia Universidad Católica de Chile, Santiago, Chile; ^10^School of Psychology, Konrad Lorenz University, Bogotá, Colombia; ^11^Facultad de Ciencias Sociales, Instituto de Investigaciones Psicológicas, Universidad de Costa Rica, San José, Costa Rica; ^12^Facultad de Psicología, Universidad de la Habana, La Habana, Cuba; ^13^Facultad de Psicología, Pontificia Universidad Católica de Ecuador, Quito, Ecuador; ^14^Céres Research Unit, TR3, Free Faculty of Arts and Humanities, Catholic Institute of Toulouse, Toulouse, France; ^15^Center for Studies and Research in Psychopathology and Health Psychology (CERPPS), Faculty of Psychology, University of Toulouse Jean Jaurès, Toulouse, France; ^16^Departamento Metodología de Ciencias del Comportamiento, Universidad Nacional de Educación a Distancia, Madrid, Spain; ^17^Departamento “Psicología Clínica y de la Salud y Metodología de Investigación”, Facultad de Psicología, University of the Basque Country, Donostia-San Sebastián, Spain; ^18^“Abby & Anna” SOGIE Lab, Eliot-Pearson Department of Child Study and Human Development, Tufts University, Medford, MA, United States; ^19^Departamento de Psicología, Instituto de Psicología, Educación y Desarrollo Humano, Universidad de la República, Montevideo, Uruguay; ^20^Department of Psychology, UCLouvain, Louvain-la-Neuve, Belgium

**Keywords:** equivalence, Spanish-speaking, exhaustion, parenting, ESEM

## Abstract

Parental burnout is a unique and context-specific syndrome resulting from a chronic imbalance of risks over resources in the parenting domain. The current research aims to evaluate the psychometric properties of the Spanish version of the Parental Burnout Assessment (PBA) across Spanish-speaking countries with two consecutive studies. In Study 1, we analyzed the data through a bifactor model within an Exploratory Structural Equation Modeling (ESEM) on the pooled sample of participants (*N* = 1,979) obtaining good fit indices. We then attained measurement invariance across both gender and countries in a set of nested models with gradually increasing parameter constraints. Latent means comparisons across countries showed that among the participants’ countries, Chile had the highest parental burnout score, likewise, comparisons across gender evidenced that mothers displayed higher scores than fathers, as shown in previous studies. Reliability coefficients were high. In Study 2 (*N* = 1,171), we tested the relations between parental burnout and three specific consequences, i.e., escape and suicidal ideations, parental neglect, and parental violence toward one’s children. The medium to large associations found provided support for the PBA’s predictive validity. Overall, we concluded that the Spanish version of the PBA has good psychometric properties. The results support its relevance for the assessment of parental burnout among Spanish-speaking parents, offering new opportunities for cross-cultural research in the parenting domain.

## Introduction

Parenthood is associated with positive emotions like joy, accomplishment, and pride in many cultures. However, evidence shows that parenting is also a complex and strained activity (e.g., Abidin, 1990; Crnic and Low, 2002; Deater-deckard, 2014). Exposure to too many stressors over long periods can lead to burnout, as has been demonstrated, when there are not enough psychological resources to deal with them in job settings (Bakker and Demerouti, 2007). The same is true in the parenting context: when the balance between parental risks and resources chronically leans to the negative side, parents are at high risk of parental burnout (Roskam et al., 2017; Mikolajczak and Roskam, 2018).

### Parental Burnout

Burnout is a disorder that is associated with chronic stress. However, the problem is not limited to factors increasing stress but to lack of resources to deal with it. This is the sustained imbalance between stress and resources that characterizes the burnout syndrome. According to this view, parental burnout is considered a singular and contextualized syndrome resulting from a chronical imbalance of risks (e.g., poor childrearing, numerous parental duties, and perfectionism) over resources (e.g., positive coparenting, external support, high emotional intelligence, etc.) in the field of parenting (Mikolajczak et al., 2018, 2019).

The demand or risk factors would intensify parental burnout. One example of such a stressor is parental perfectionism, which has been proven to be detrimental to parental burnout. A study conducted by Sorkkila and Aunola (2020) involving more than 1,500 Finnish parents determined that socially prescribed perfectionism, demanded by significant others in the individual environment through imposition of very high-performance expectations and the tendency to harshly judge the results (Flett and Hewitt, 2002), constitutes a risk factor. Similar results were obtained by Kawamoto et al. (2018) in a study settled in Japan with 1,200 participants, in which parental perfectionism was a strong stressor contributing to parental burnout. Resources, on the other hand, would lower parental burnout. For example, emotional competences, as the ability to process information about one’s own emotions and those of others, enabling people to pay attention, use, understand, and manage their emotions (Mayer and Salovey, 1997) plays a protective role for parental burnout. The study of Lin et al. (2021) with two independent samples (i.e., Poland and Belgium) shed light on the key role played by emotional competences buffering the effect of parental perfectionism on parental burnout. Mikolajczak and Roskam (2018) proposed a Balance between Risk and Resources (BR^2^) theory in which “parental burnout develops when parental resources are insufficient to meet the demands (whatever they are)” (p. 2). While resources and risks play opposite roles with regard to parental burnout, the absence of one of them does not equate to the presence of the other. For example, the absence of denigration by the partner does not mean that the parent has the support of the partner. And a risk does not necessarily have to be offset by the corresponding resource but can be offset by one or more other resources. For example, the lack of partner support may be compensated for by social support or by the children’s autonomy. Parental burnout is thus the result of an imbalance between several factors and of risk not compensated by sufficient resources to cope with it. In a two-wave longitudinal study conducted over 900 parents, results highlighted that the BR^2^ framework predicted parental burnout (Mikolajczak and Roskam, 2018).

Parental exhaustion is the core symptom of parental burnout. Parents experience tiredness at every aspect of their daily routine of attending to their children. The emotional drain is such that they often see themselves reaching their limits. Another symptom is the emotional distancing from their children. Parents also progressively reduce their involvement in parenting, while their relationship with their children suffers; interactions decrease and are limited mostly to functional/instrumental aspects at the detriment of emotions. Another symptom is the loss of feeling of accomplishment as a parent: being fed up with parenting, parents can no longer stand their role, and time with their children is no longer appreciated. All these symptoms and states represent a contrasting view of the parental self, i.e., previous feelings toward parenting (Roskam et al., 2018).

Parental burnout should not be regarded as typical parental stress (Roskam et al., 2017; Kawamoto et al., 2018; Lebert-Charron et al., 2018; Van Bakel et al., 2018; Brianda et al., 2020). It results from tremendous parental stress, as a long-lasting response (Mikolajczak and Roskam, 2018). In a cross-cultural study gathering 42 countries across the world, the prevalence of parental burnout varied from 0 to more than 8% (Roskam et al., 2021), even after controlling for sample size and sex imbalance. Poland (7%), Belgium (7.9%), and the United States (8.4%) were the countries with the highest levels of parental burnout. It is also not comparable to other forms of burnout, such as job burnout, given that these two constructs have low to moderate correlations (Roskam et al., 2017; Kawamoto et al., 2018; Van Bakel et al., 2018). Finally, although parental burnout can lead to depression, it is not the same as depression (Kawamoto et al., 2018; Van Bakel et al., 2018; Sánchez-Rodríguez et al., 2019). Parental burnout is a particular syndrome, which is context-specific (Mikolajczak et al., 2020).

The burnout syndrome matters because is associated with numerous negative outcomes, e.g., psychopathology symptoms (Mesman and Koot, 2001). In the parenthood field, the burnout syndrome has far-reaching consequences. Recent studies suggest that it has harmful effects for the parent, in particular suicidal thoughts. Suicidal ideations are associated with profound feelings of hopelessness. Individuals do not believe that their problems could be solved, and consequently, there is no way out from suffering. Because the parental role can never be left behind and parents can never resign, medium to large associations between parental burnout, and escape and suicidal ideations have been found (Mikolajczak et al., 2018). Parental burnout also has deleterious consequences on children (Mikolajczak et al., 2018, 2019). Medium to large associations between parental neglect and parental violence toward children and parental burnout were reported (Mikolajczak et al., 2018). Burned-out parents are so exhausted that they no longer take care of their children, despite understanding their needs. With emotional distance and loss of accomplishment in parenting, verbal or physical violence toward children also increases (Blanchard et al., 2020; Hansotte et al., 2021). The deleterious consequences of burnout highlight the need for reliable measures in each specific context, either job or parenting, as appropriate.

### Assessment of Parental Burnout

Pelsma et al. (1989) introduced parental burnout measurement by advocating that the Maslach Burnout Inventory (MBI, Maslach et al., 1986) might be a good foundation for constructing a measure of parental burnout. His intuition was partially supported, and he did not pursue the investigation. It took around 20 years until his work gained recognition. In 2007, Norberg, who was then working at a children’s hospital, noticed that parents of severely ill children presented burnout symptoms. Norberg’s team then explored this issue over the next 7 years using the Shirom–Melamed Burnout Questionnaire (SMBQ, Melamed et al., 1992). The SMBQ is a 14-item questionnaire consisting of ten context-free items and four job-related items. Although preliminary support of the notion of parental burnout could be found in these studies, skeptics could argue that having an ill child made parents at risk to develop job burnout.

Roskam et al. (2017) were the first authors to validate a specific instrument for assessing parental burnout. They were the first to adapt Maslach Burnout Inventory© (MBI, Maslach et al., 1986) so that all chosen items referred specifically to the parenting domain. The “depersonalization” subscale did not work in the parental context and the authors replaced it with an emotional distancing subscale. Their development and validation studies resulted in the Parental Burnout Inventory (PBI, Roskam et al., 2017), a 22-item measure of parental burnout encompassing three factors: parental exhaustion, emotional distancing from the children, and reduced parental effectiveness, alongside lack of fulfillment. The approach used to develop the Parental Burnout Inventory was deductive since it was based on the MBI. This resulted in unanswered questions about the three dimensions they proposed to depict parental burnout.

Roskam et al. (2018) used the inductive method to deepen the conceptualization and measurement of parental exhaustion. For this purpose, colleagues who were oblivious of the aforementioned three-dimensional structure analyzed testimonies from a large sample of Anglo-Saxon and francophone exhausted parents, based on the Interpretative Phenomenological Analysis (IPA) (Hubert and Aujoulat, 2018). From the IPA, 50 items transpired which were reduced to 23 items after factor analysis, thus resulting in the Parental Burnout Assessment (PBA). This questionnaire covers four dimensions, two of which are identical to those of the PBI, i.e., Emotional Exhaustion in the parental role and Emotional Distancing from one’s children, and the other two distinct, i.e., Feelings of Being Fed Up, and Contrast in parental self. Because of its theoretical background, psychometric properties, and open access, the PBA is considered a quality instrument for assessing Parental Burnout.

### Measurement Equivalence Across Countries

One of the main purposes of cross-cultural research is to compare psychological constructs between distinct groups. A measure that has been deemed psychometrically sound in one cultural setting is often assumed to be translatable for use in another cultural context, but this assumption may be misleading and needs to be formally tested. Currently, the only evidence for the cross-cultural equivalence of the PBA construct has been found between French- and English-speaking samples in Western countries (Roskam et al., 2018). This does not exclude the possibility that parental burnout may vary, both structurally and expressively, across countries or cultures. An important challenge in the large-scale assessments commonly used in cross-cultural research is to verify that the collected self-reported data measure the object of interest in the same way, irrespective of the macro-context. Due to differences in language and cultural response biases, participants interpret certain words in different ways. As a result, their understanding of the intended meaning of a question changes, and respondents may attribute different meanings to an instrument, undermining the comparability of constructs across cultures (Markus and Kitayama, 1991; Rutkowski and Svetina, 2014).

### The Current Research

The current research aims to evaluate the psychometric properties of the Spanish version of the PBA. In order to test the validity of its internal structure, we first conducted an exploratory analysis using an Exploratory Structural Equation Modeling (ESEM) framework (Marsh et al., 2010). ESEM is an interesting alternative approach to classical Confirmatory Factorial Analysis (CFA) commonly used in previous studies (e.g., Aunola et al., 2020; Sodi et al., 2020; Szczygieł et al., 2020; Roskam et al., 2021). In CFA, the influence exerted on a given item by dimensions other than the one(s) to which this item belongs can be hidden (Marsh et al., 2010). In previous PBA validation studies, the presence of high inter factorial correlations (>0.90) was not explicitly addressed, which might represent a threat to multicollinearity (Brown, 2015). When overlaps occur, interpreting factors as independent entities may be no longer appropriate. Another reason for implementing ESEM is that, from a conceptual point of view, the good fit indices found for a culturally-invariant model in different countries do not guarantee that this model is the best representation of parental burnout in each country.

**FIGURE 1:**
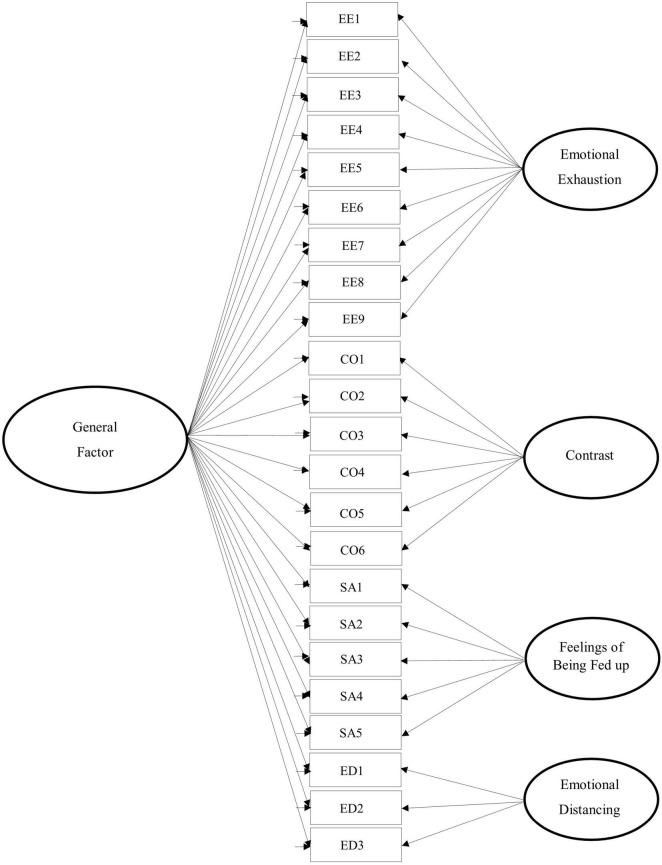
The conceptual bifactor model of parental burnout.

For these reasons, we examined whether a bifactor model of the PBA could be applied to the sample of Spanish-speaking parents. Bifactor modeling assumes that the correlations between factors are explained by the presence of a general factor (Reise, 2012). The analysis simultaneously models the influence of specific (orthogonal) factors and a general factor on a set of items. This technique provides even more solid evidence regarding the relevance of a general factor (Parental Burnout in the current study) compared to a second-order hierarchical model. Hence, bifactor modeling in ESEM tests the direct influence of the general factor on the items, whereas second-order hierarchical modeling tests the influence of the general factor on the items indirectly through the first-order factors (Canivez, 2016). Figure 1 depicts the conceptual model of the general measure of parental burnout.

We then tested measurement invariance across genders and countries (Argentina, Chile, Costa Rica, Cuba, Ecuador, Peru, Spain, and Uruguay), and we examined mean differences. Based on previous research (Pérez-Díaz and Oyarce Cádiz, 2020; Roskam and Mikolajczak, 2020), parental burnout was expected to be higher among mothers than fathers. In an exploratory way, we tested the mean differences across countries regarding the total score of parental burnout. Finally, to obtain evidence of validity for the relationship of the PBA with other theoretically related variables, predictive validity was estimated by testing associations between parental burnout and three specific effects of this construct, i.e., escape and suicidal ideations, parental neglect, and parental violence toward one’s children, in two countries displaying different mean levels of parental burnout, i.e., Chile and Ecuador.

## General Method

### Overview

The two studies were part of the International Investigation of Parental Burnout (IIPB), a 42-country study of parental burnout around the globe (Roskam et al., 2021). An institutional review board provided ethical clearance for the research in each country [i.e., Spain (Bioethics Committee from The National Distance Education University); Perú (The Research Committee of the San Martín de Porres University); Chile (Ethics Committee from the Autonomous University of Chile, 71–18); Uruguay (Ethics Committee of the Faculty of Psychology of the University of the Republic); Ecuador (Institutional Review Commission of the Catholic University of Ecuador); Cuba (Research Ethics Committee of the Faculty of Psychology of the University of Havana, Dossier N-3); and Costa Rica (Scientific Ethics Committee of the University of Costa Rica, N93 VI-1071-2018)]. Note that in Argentina, no ethical clearance was required for the research.

Both studies complied with APA ethical standards regarding the treatment of human subjects. The inclusion criterium for the IIPB study was having at least one child still living at home. In the current study, we introduced two additional criteria for the inclusion of the participants. Parents were required to have been born and resided in the same country where the data collection took place. Parents signed an informed consent allowing withdrawal at any moment. We also assured participants that the data would remain anonymous.

In Study 1, a common survey, made up of sociodemographic characteristics and the measure of parental burnout (i.e., PBA), was used in the eight included countries. The Spanish version of the PBA was produced using translation/back-translation procedures by the Hispanic members of the IIPB consortium. Minor country-specific language modifications were implemented. Participants completed the survey online (in Argentina, Chile, and Ecuador), on paper (in Cuba and Uruguay), or both (in Costa Rica, Peru, and Spain). The online version was administered using the Qualtrics platform. Information about the study was disseminated through social media (Facebook, LinkedIn, and Instagram), by word of mouth or through an official email invitation sent by the researchers. For the paper-and-pencil version, participants were recruited through parents’ associations, as well as private and public schools. The data were mostly collected in urban zones of the capital in each country.

Study 2 was based on a second data collection in Chile and Ecuador. The survey comprised measures of parental burnout, escape, suicidal ideations, parental neglect, and parental violence toward one’s children. All participants completed the survey online using the Qualtrics platform. We implemented a snowball sampling technique. As in Study 1, the data were primarily collected in urban zones of the capital in each country.

In the section “General Method” as well as in the “Method” sections of Study 1 and Study 2, we reported how we made sure that the statistical analyses were appropriate for the sample sizes in each participating country, all data inclusion criteria (all being established prior to data analysis), the measures used in the two studies, and all the statistical analyses we performed. We reported exact *p* values, effect sizes, and 95% confidence intervals for inferential analyses.

### Data Analyses

In Study 1, we used a bifactor ESEM model to evaluate how adjusted the parental burnout measurement model is. The assumptions of the bifactor model are that the specific factors are orthogonal to each other and that the presence of a general factor explains better the correlations between them. We preliminarily tested the bifactor model structure of the PBA in each country sample independently. Following that, we performed ESEM to inspect the bifactor model on the pooled sample.

Several items showed deviation from normality, as their skewness and kurtosis proved. This is expected, as mental health indicators usually feature an asymmetric distribution. A positively skewed distribution for these parental burnout items is therefore conceptually sound. We used maximum likelihood estimation with robust (Huber-White) standard errors and a scaled test statistic (MLR) as the estimation method while relying on standard fit indices to ascertain the adequacy of the Bifactor ESEM model: the Satorra-Bentler scaled chi-square statistics (S-Bχ2; Satorra and Bentler, 1991), the Tucker-Lewis index (TLI), the comparative fit index (CFI), the root mean square error of approximation (RMSEA), and the standardized root mean square residual (SRMR). Should the CFI and TLI values reach 0.90 or more, they are considered adequate, and values of RMSEA and SRMR ≤ 0.8 are preferred (Hu and Bentler, 1999). These analyses were executed with the software Mplus version 7 (Muthén and Muthén, 1998-2015).

The fit indices do not report on the strength of the general factor. Thus, complementary indicators were implemented to evaluate the strength of the general factor, i.e., the general hierarchical omega (ω_h_; Zinbarg et al., 2006), and the explained common variance (ECV; Sijtsma, 2009). Values greater than 0.70 and 0.60, were respectively expected (Reise et al., 2013), while values greater than 0.30 for the hierarchical omega per dimension (ω_hs_) meant the interpretability of the factors (Smits et al., 2015).

When testing for measurement invariance, we used a set of models where constraints were incrementally introduced through a stepwise multiple group analysis (MG-ESEM). First, the parental burnout model was assessed for configural invariance. Second, item factor loadings were tested in a metric invariance model. Third, scalar invariance was verified with the intercepts set as equal across groups. Finally, error variances were constrained to be equal across groups. A CFI decrease within the established limits (ΔCFI ≤ -0.01), RMSEA and SRMR indices within certain thresholds (ΔRMSEA ≤ 0.015; ΔSRMR ≤ 0.030) confirms measurement invariance (Cheung and Rensvold, 2002; Chen, 2007). Note that measurement invariance across genders was estimated on the pooled sample, rather than in each of the countries because of the small sample size of fathers, and to a lesser extent, of mothers in some countries.

To evaluate gender and country-related PBA mean differences, the latent mean of the reference groups (Chile for countries and fathers for gender) was set to zero within the framework of the scalar invariance model. In this sense, the statistical significance of the mean difference of each sample compared to the reference group was assessed in each analysis, either across countries or genders. Therefore, a latent mean difference (LM_diff_) greater than zero indicated that the comparison group had a higher mean than that of the reference group (Brown, 2015). To calculate whether the latent means of the comparison group differed significantly from the baseline, we calculated critical ratios (CRs) by dividing the estimate by its standard error. When the CR was above 1.96, the difference was statistically significant. To calculate whether the latent mean of the comparison group differed significantly from the baseline, we calculated critical ratios (CRs) by dividing the estimate by its standard error. When the CR was above 1.96, the difference was statistically significant (Byrne, 2013). We supplemented the comparisons with Cohen’s d measure of effect size with confidence intervals, where values less than 0.20 indicated an insignificant effect size, between 0.20 and 0.50 a small magnitude, between 0.50 and 0.80, moderate; and greater than 0.80, large (Ferguson, 2009).

In Study 2, the predictive validity of the PBA was estimated using bivariate correlations between the general factor of the PBA on the one hand, and escape and suicidal ideations, parental neglect, and parental violence toward one’s children on the other hand. Confidence intervals for correlation coefficients were calculated.

We estimated the reliability of the scales used in the current research by computing the Cronbach’s alpha (α) and McDonald’s omega coefficient (ω). A hierarchical omega (ω_h_) was also calculated within the framework of the bifactor model (Rodriguez et al., 2016). While Cronbach’s alpha is based on the correlations between test items, model-based coefficients like omega are rather based on the latent structure of the test. Cronbach’s alpha can be overestimated because all the shared variance between items counts toward the coefficient. In contrast, omega bypasses this caveat, due to estimating reliability on the factorial loads, thus for the construct in the analysis.

## Study 1

### Participants

The initial sample (*N* = 2,502) encompassed participants from Argentina, Chile, Costa Rica, Cuba, Ecuador, Peru, Spain, and Uruguay. Of these, 1,869 (74.7%) were women and 633 (25.3%) were men. Their age ranged from 17 to 78 (*M* = 38.03, *SD* = 8.49). However, since one of the aims of the study is a multigroup analysis and due to the absence of convergence, three samples were excluded (Argentina, Cuba, and Uruguay after initial processing). Similarly, Ecuador’s data from Study 1 were replaced by Study 2 due to the larger sample size.

With this final sample (*N* = 1,979) the majority of participants were women (1,588, 80.2%). Their age ranged from 17 to 78 years (*M* = 38.43, *SD* = 8.09). The number of children ranged from 1 to 9 (*M* = 1.79, *SD* = 0.88). Table 1 displays demographic information for participants in each country.

**TABLE 1:** Demographic characteristics of the samples in Study 1.

	*N*	*n*_*Women*_	*n*_*Men*_	Mean age (*SD*)	Mean age_Women_ (*SD*)	Mean age_Men_ (*SD*)	Educational level (years of education)			Number of children
							12 years (compulsory education)	17 years (university degree)	>18 years (postgraduate degree)	Mean [Range]
Argentina	176	118	58	39.95 (9.88)	40.43 (9.23)	38.97 (11.04)	32 (18.1%)	79 (44.6%)	65 (37.3%)	2.35 [1–8]
Chile*	718	641	77	36.89 (6.01)	36.49 (5.70)	40.25 (7.38)	25 (5.2%)	273 (41.9%)	420 (52.9%)	1.79 [1–9]
Costa Rica*	234	14	94	37.68 (8.15)	35.66 (6.98)	40.69 (8.85)	29 (16.6%)	102 (41.5%)	103 (41.9%)	1.59 [1–5]
Cuba	240	137	103	40.04 (10.24)	39.96 (10.32)	40.16 (10.19)	143(59.6%)	71 (29.6)	26 (10.8%)	1.70 [1–4]
Ecuador**	146	102	44	32.45 (7.51)	32.27 (7.34)	32.86 (7.96)	9 (6.2%)	69 (47.3%)	68 (46.6%)	1.64 [1–5]
Peru*	368	265	103	40.22 (10.84)	38.97 (9.89)	43.815 (12.34)	99 (30.6%)	144 (57.8%)	125 (11.6%)	1.93 [1–5]
Spain*	333	287	46	41.10 (7.86)	40.80 (7.80)	42.98 (8.09)	104(32.4%)	133 (43.1%)	96 (24.5%)	1.74 [1–6]
Uruguay	287	179	108	35.02 (6.42)	34.23 (5.80)	36.36 (7.16)	150(50.7%)	90 (33.1%)	47 (16.2%)	1.60 [1–6]

*Denotes data sets involved in study 1 and **For the case of Ecuador we used dataset of study 2.

### Measures

#### Parental Burnout

Parental burnout was assessed by the Parental Burnout Assessment (PBA, Roskam et al., 2018), a 23-item self-report. The instrument encompasses four dimensions: Emotional Exhaustion [nine items; e.g., “I have zero energy for looking after my child(ren)”], Contrast (six items; e.g., “I’m ashamed of the parent that I’ve become”), Feelings of Being Fed Up (five items; e.g., “I feel like I can’t cope as a parent”), and Emotional Distancing [three items; e.g., “I’m no longer able to show my child(ren) how much I love them”]. Items are rated on 7-point Likert scales: never (0), a few times a year or less (1), once a month or less (2), a few times a month (3), once a week (4), a few times a week (5), every day (6). Cronbach’s alphas reported in the original PBA version were high, ranging from 0.77 to 0.94. Spanish PBA scale can be found in Supplementary Material.

### Results

#### Parental Burnout Assessment Item Description and Factor Analysis

The mean of the items was *M*_*mean*_ = 1.09 (range: 0.38–2.64) and the standard deviation was *SD_*mean*_* = 1.44 (range: 1.04–1.99). Several items were positively skewed [*M*_*Sk*_ = 1.84, range (0.22–3.42)] and showed high kurtosis [*M*_*k*_ = 3.55, range (−1.27 to 12)]. Methods of estimation which do not require normality were applied, as mentioned in the “Data Analyses” section. Due to the complexity of the ESEM approach and the number of parameters to be estimated, the model could not converge in three samples, so they were removed from the analyses.

The bifactor ESEM model was estimated in the five remaining samples and the pooled sample (*N* = 1,979). Fit indices were good, as displayed in Table 2. Moreover, the magnitude of the descriptive indicators associated with the bifactor analysis (ECV, ω_h_, and ω_hs_) demonstrated the strength of the general factor across countries and genders (see Table 3). In addition, as shown in Table 3, the standardized factor loadings ranged from 0.57 to 0.81 for the general factor of Parental Burnout in the pooled sample (*N* = 1,979).

**TABLE 2:** Bifactor ESEM model and reliability per country and gender.

						GF		EX	CO	SA	ED		
	*S-B*χ^2^ [148]	*CFI*	*TLI*	*RMSEA*	*SRMR*	*ECV*	ω*h*	ω*hs*	ω*hs*	ω*hs*	ω*hs*	ω	α
				[90% IC]									[95% IC]
**Countries**													
Chile	410.519**	0.964	0.939	0.050	0.017	0.823	0.930	0.103	0.181	0.061	0.176	0.900	0.961
				[0.044–0.055]									[0.0957–0.964]
Ecuador	317.956**	0.916	0.857	0.068	0.025	0.837	0.944	0.016	0.203	0.040	0.147	0.894	0.957
				[0.060–0.077]									[0.952–0.963]
Costa Rica	257.012**	0.965	0.940	0.056	0.020	0.821	0.926	0.207	0.040	0.107	0.087	0.905	0.963
				[0.044–0.067]									[0.956–0.968]
Perú	302.643**	0.927	0.874	0.053	0.025	0.803	0.913	0.046	0.269	0.065	0.161	0.869	0.942
				[0.045–0.062]									[0.934–0.949]
Spain	256.985**	0.967	0.944	0.047	0.019	0.811	0.932	0.012	0.320	0.060	0.128	0.895	0.957
				[0.037–0.057]									[0.952–0.963]
Total Sample	542.044**	0.976	0.958	0.037	0.014	0.829	0.929	0.070	0.231	0.065	0.122	0.958	0.958
				[0.033–0.040]									[0.955–0.960]
**Gender**													
Fathers	249.703**	0.949	0.913	0.042	0.025	0.750	0.882	0.109	0.332	−0.147	0.109	0.862	0.929
				[0.033–0.051]									[0.920–0.937]
Mothers	509.523**	0.974	0.956	0.039	0.015	0.830	0.931	0.065	0.229	0.055	0.134	0.894	0.959
				[0.036–0.043]									[0.956–0.961]

S-B χ2, Satorra Bentler chi-squared; CFI, Comparative Fit Index; TLI, Tucker–Lewis index; RMSEA, Root Mean Square Error of Approximation; SRMR, Standardized Root Mean Square Residual; ECV, Explained Common Variance; GF, General Factor; EX, Exhaustion; CO, Contrast; SA, Saturation; DE, Emotional Distancing; ωh, General Omega hierarchical; ωhs, Omega hierarchical per dimension; α, Cronbach’s alpha. **p < 0.01.

**TABLE 3:** Factor loadings of the bifactor ESEM model in the pooled sample.

	GF	EX	CO	SA	ED
EX1	0.574	**0.453**	−0.053	−0.160	−0.134
EX2	0.736	**0.435**	−0.007	−0.025	−0.143
EX3	0.696	**0.381**	0.025	−0.003	0.008
EX4	0.698	**0.235**	−0.038	−0.057	0.075
EX5	0.767	**0.097**	0.031	0.347	−0.083
EX6	0.772	**0.235**	−0.074	−0.062	−0.010
EX7	0.780	**0.096**	−0.105	−0.036	0.010
EX8	0.802	**0.012**	−0.138	−0.122	0.030
EX9	0.813	**−0.047**	0.005	−0.035	0.122
CO1	0.616	0.283	**0.244**	0.067	0.027
CO2	0.652	0.234	**0.400**	0.090	0.229
CO3	0.700	0.075	**0.399**	0.067	0.167
CO4	0.713	−0.214	**0.492**	0.040	−0.058
CO5	0.663	−0.194	**0.466**	−0.037	−0.127
CO6	0.726	−0.133	**0.374**	0.001	0.153
SA1	0.712	0.037	0.080	**0.520**	−0.017
SA2	0.743	0.344	−0.110	**-0.082**	−0.102
SA3	0.619	−0.079	0.035	**0.125**	−0.009
SA4	0.810	0.172	−0.098	**0.022**	−0.073
SA5	0.732	−0.152	0.066	**0.437**	−0.071
ED1	0.606	−0.104	0.084	−0.006	**0.249**
ED2	0.581	−0.219	0.284	0.022	**0.241**
ED3	0.665	−0.109	0.129	−0.109	**0.347**

*Factor loadings in bold belong to the theoretical factor. GF, General Factor; EX, Exhaustion; CO, Contrast; SA, Saturation; ED, Emotional Distancing.*

#### Measurement Invariance of Parental Burnout Bifactor Exploratory Structural Equation Modeling Model Across Countries

We used the Chilean sample as the reference group. As shown in Table 4, the fit indices support configural invariance. Under the constraint of equal factor loadings, the model fit did not significantly worsen, providing evidence for metric invariance. In the next step, when intercepts were constrained, scalar invariance was evidenced. Finally, error invariance was attained.

**TABLE 4:** Goodness-of-fit indices of measurement invariance of the PBA bifactor ESEM model across countries and gender.

	*S-B*χ^2^	*df*	*RMSEA*	Δ*RMSEA*	*CFI*	Δ*CFI*	*SRMR*	Δ*SRMR*
**Across Countries**								
Configural	1.602.712	740	0.054		0.953		0.021	
Metric	1.767.875	1100	0.039	−0.014	0.963	−0.01	0.040	0.019
Scalar	1.980.609	1172	0.042	0.003	0.956	−0.007	0.042	0.002
Error	2.168.826	1264	0.043	0.001	0.95	−0.006	0.053	0.011
**Across Gender**								
Configural	769.355	296	0.040		0.969		0.017	
Metric	846.208	386	0.035	−0.005	0.970	0.001	0.028	0.011
Scalar	890.556	404	0.035	0.000	0.968	−0.002	0.029	0.001
Error	NC	NC	NC	NC	NC	NC	NC	NC

*RMSEA, Root Mean Square Error of Approximation; CFI, Comparative Fit Index; SRMR, Standardized Root Mean Square Residual; S-Bχ^2^, Satorra Bentler chi-squared; df, Degrees of freedom; ΔRMSEA, Root Mean Square Error of Approximation Difference Test;ΔCFI, Comparative Fit Index Difference Test; ΔSRMR, Standardized Root Mean Square Residual Difference Test; NC, No Convergence.*

#### Measurement Invariance of Parental Burnout Bifactor Exploratory Structural Equation Modeling Model Across Genders

We used fathers as the reference group. According to the variation observed in the CFI, RMSEA, and SRMR fit indices, an acceptable degree of factorial invariance was found up to the scalar level (see Table 4).

#### Mean Differences Across Countries

Latent means, standard errors, effect sizes, and latent means comparisons across countries are presented in Table 5. Significant differences in the level of Parental Burnout were found between the five Spanish-speaking countries. In particular, Chile had the highest parental burnout score, whereas Peru had the lowest.

**TABLE 5:** Comparisons across reference and comparison countries.

	*LM*_*diff*_	*SE*	*CR*	*d* (95% CI)
Costa Rica	−0.191	0.090	−2.122*	0.28 [0.13–0.43]
Ecuador	−0.315	0.097	−3.247*	0.32 [0.19–0.45]
Peru	−0.506	0.102	−4.961*	0.45 [0.32–0.57]
Spain	−0.142	0.077	−1.844	0.18 [0.05–0.31]

LM, Latent means; LM_diff_, difference relative to the mean of this referent group; * denotes Critical Ratio value larger than 1.96 indicating statistically significant differences in the LM; d, Cohen’s d; CI, confidence interval.

#### Mean Difference Across Genders

Parental burnout general latent mean for fathers was set to be 0, and as expected, we found gender-related differences in parental burnout. Mothers displayed higher score than fathers on the Parental Burnout general factor (*LM*_*diff*_ = 0.545, *SE* = 0.046), with a significant Critical Ratio (CR = 11.85) but with a small effect size (*d* = 0.32).

#### Reliability Indexes

The coefficients found for the scales used in the current study were high (see Table 2).

## Study 2

### Participants

The total sample (*N* = 1,171) encompassed participants from Chile (*N* = 845) and Ecuador (*N* = 326). Of these, 1,044 (89.2%) were women and 125 (10.8%) were men. Their age ranged from 19 to 66 (*M* = 37.99, *SD* = 7.08). The number of children living at home ranged from 1 to 8 (*M* = 1.81, *SD* = 0.87); the mean age of the oldest child was 9.51 (*SD* = 7.40), and the mean age of the youngest child was 6.03 (*SD* = 6.30). Table 6 shows the demographic information for participants in each country.

**TABLE 6:** Demographic characteristics of the samples in study 2.

	*N*	*n*_*Women*_	*n*_*Men*_	Mean age (*SD*)	Mean age_Women_ (*SD*)	Mean age_Men_ (*SD*)	Educational level (years of education)			Number of children
							12 years (compulsory education)	17 years (university degree)	>18 years (postgraduate degree)	Mean [Range]
Chile	845	789	54	38.08 (6.70)	37.94 (6.62)	40.04 (7.60)	29 (3.4%)	321 (38.08%)	493 (58.52%)	1.78 [1–5]
Ecuador	326	255	71	37.79 (7.99)	37.09 (7.46)	40.32 (9.27)	41 (11.9%)	128 (40%)	157 (48.1%)	1.87 [1–8]

### Measures

#### Parental Burnout

Parental burnout was measured using the Spanish version of the PBA, as previously presented and described in Study 1.

#### Escape and Suicidal Ideations

Escape and suicidal ideations were evaluated by a seven-item questionnaire (e.g., “I have thoughts about leaving my family”) rated on a 7-point scale of frequency, i.e., never (1), less than once a month (2), about once a month (3), two or three times a month (4), once a week (5), several times a week (6), every day (7) (Mikolajczak et al., 2018). We obtained the escape and suicidal ideations score by adding the seven item scores. In the current study, the Cronbach’s alpha was 0.83 [0.81, 0.84], and Omega’s coefficient (ω) was 0.86 [0.80, 0.90].

#### Parental Neglect

Neglectful behavior toward one’s children was evaluated with the Parental Neglect Scale (Mikolajczak et al., 2018), a 17-item questionnaire (e.g., “I don’t care about my child’s schooling and future”) rated on an 8-point scale of frequency, i.e., never (1), less than once a month (2), about once a month (3), a few times a month (4), once a week (5), several times a week (6), every day (7), and several times a day (8). The parental neglect score was obtained by adding the 17 item scores. In the current study, Cronbach’s alpha was 0.88 [0.87, 0.89], and Omega’s coefficient (ω) was 0.88 [0.86, 0.91].

#### Parental Violence

Parental Violence was evaluated with the Parental Violence Scale (Mikolajczak et al., 2018), a fifteen-item questionnaire targeting verbal violence [e.g., “I say things to my children that I then regret (insults, ridiculous nicknames, etc.)”], physical violence (e.g., “I sometimes spank or slap my child”), and psychological violence (e.g., “I tell my children that I will abandon them if they are not good”). Items are rated on an 8-point scale of frequency, i.e., never (1), less than once a month (2), about once a month (3), a few times a month (4), once a week (5), several times a week (6), every day (7), and several times a day (8). The parental violence score was obtained by adding the 15 item scores. In the current study, Cronbach’s alpha was 0.86 [0.85, 0.88], and Omega’s coefficient (ω) was 0.86 [0.80, 0.90].

### Results

Table 7 shows the correlations between the total score of the PBA on the one hand, and escape and suicidal ideations, parental neglect, and parental violence toward one’s children on the other hand. We found evidence for the predictive validity of the PBA – indeed, we perfectly replicated the medium to large associations previously reported with both French and English-speaking samples of parents (Mikolajczak et al., 2018). In particular, we found large correlations between parental burnout and escape and suicidal ideations. We also found medium correlations between parental burnout and both parental neglect and parental violence.

**TABLE 7:** Means, standard deviations, and Pearson correlations with confidence intervals between PBA general factor and measures of escape and suicidal ideations, parental neglect, and parental violence toward one’s children.

	*M*	*SD*	Range	1	2	3	4
1. Escape, suicidal ideations	9.79	4.48	7–40	−	0.33**	0.34**	0.56**
					[0.28, 0.38]	[0.29, 0.39]	[0.52, 0.60]
2. Parental neglect	28.54	12.74	17–136		−	0.59**	0.45**
						[0.55, 0.63]	[0.40, 0.49]
3. Parental violence	21.86	8.64	15–120			−	0.33**
							[0.28, 0.38]
4. PBA general factor	28.67	25.60	0–133				−

M, Means; SD, Standard Deviations. Values in brackets indicate 95% confidence interval for each correlation, **p < 0.001.

## General Discussion

This research aimed to evaluate the psychometric properties of the Spanish version of the PBA. We collected data in eight Spanish-speaking countries (i.e., Argentina, Chile, Costa Rica, Cuba, Ecuador, Peru, Spain, and Uruguay). In Study 1, we analyzed the internal structure of the PBA using the ESEM approach considered as the combination of the best components of CFA and conventional EFA. Previous research supported a general factor of the PBA through CFA and second-order hierarchical factor modeling (e.g., Matias et al., 2020; Mousavi et al., 2020; Sodi et al., 2020; Szczygieł et al., 2020; Roskam et al., 2021). By means of bifactor modeling and ESEM, the findings hereby presented complements previous studies on the suitability of the Parental Burnout general score for accurately assessing parental burnout (Canivez, 2016). We further examined the invariance of the bifactor ESEM model across countries (i.e., Chile, Costa Rica, Ecuador, Peru, and Spain) and gender. Moreover, we estimated the reliability of the PBA general factor across countries and tested the mean differences across gender and countries. The main conclusion from Study 1 is that the findings from the five participating countries provide evidence on the validity of the PBA as a reliable and equivalent measure of parental burnout for Spanish-speaking countries. In Study 2, we evaluated the predictive validity of the PBA in an independent sample of parents from Chile and Ecuador, two countries displaying different mean levels of parental burnout. The association between parental burnout and the three specific consequences (i.e., escape and suicidal ideations, parental neglect, and parental violence toward one’s children), were therefore tested.

The examination of the factorial structure of the PBA revealed that bifactor ESEM modeling satisfactorily fitted the data. Moreover, the PBA general factor showed good internal consistency (from 0.87 to 0.94), similar to what the pioneering study of Roskam et al. (2018) reported (from 0.77 to 0.94). Additionally, these results are comparable to the reliability of other parental burnout instruments such as the PBI conducted in Japan (from 0.84 to 0.89; Kawamoto et al., 2018) and the Netherlands (from 0.87 to 0.92; Van Bakel et al., 2018). Moreover, the scalar invariance attainment suggests we can safely compare raw scores of the PBA between countries. It is worth noting that we discovered significant statistical differences in the mean scores. For instance, the Chilean sample scored higher in parental burnout than the remaining countries, followed by the Spanish sample, which also scored above average. One possible explanation for such divergent findings relates to the influence of cultural differences.

As Hofstede (2001) illustrates, countries/cultures differ according to their position on the individualism/collectivism continuum. Therefore, individualist societies emphasize individual needs over those of the group, and people usually think of themselves as autonomous, independent, with a strong sense of self-efficacy (Triandis, 1995; Darwish and Huber, 2003). On the other hand, their competitiveness, driven by the need for self-realization, has been shown to lead to high social anxiety and affects social relationships (Park and Crocker, 2005). Because individualism/collectivism is a continuum, the position of the countries that are not on the extreme ends is–by definition – relative. For instance, although Spain is certainly more collectivist than several other European countries (e.g., France), it is nonetheless more individualist than most Latin-American countries (Roskam et al., 2021).

As mentioned earlier, in the so-called individualist countries (in this case, Spain), respondents take care of their immediate family, albeit they do not usually rely on their close network. In contrast, respondents from collectivist countries (such as Peru) actively seek support not only from the extended family (e.g., grandparents) but also from their social networks, such as friends. Since parents in individualist societies are expected to perform most chores by themselves, this may shed some light on the higher scores of parental burnout in such societies. Furthermore, we found another idiosyncrasy in the Chilean sample. Although this society remains highly family-oriented, individualist values are on the rise, especially among the younger population (Martinez et al., 2006). With modern developments such as globalization, it is likely that parents in traditionally collectivist cultures are gradually becoming more individualist (Bush, 2000; Ingoldsby et al., 2003; Bush and Peterson, 2013). These results suggest that cultural factors could be at play, although future studies are required to clarify these discrepancies.

The results show a significant difference between genders, with mothers exhibiting higher parental burnout scores than fathers. This finding is consistent with previous studies (Pérez-Díaz and Oyarce Cádiz, 2020; Roskam and Mikolajczak, 2020). A possible explanation lies in mothers’ greater involvement in the upbringing and daily care of children: a large-scale study conducted by Meier et al. (2016) showed that the ratio of tedious tasks, such as cooking and childcare, to recreational tasks (i.e., involving leisure time with their children), is higher among mothers than fathers. A recent study conducted in 40 countries by Roskam et al. (2022), also showed that parental burnout was higher among mothers who have egalitarian values and who are raising their children in a country where gender equality is high in domains such as access to health care or employment. These mothers would have high expectations of equality in the domain of parenting as well, whereas in this domain specifically, the vast majority of tasks related to childcare and childrearing are still assumed by mothers. Political and social initiatives should be taken to support gender equality in the domain of parenting, as in other ones.

The main conclusion of Study 2 is that the PBA has very good predictive validity, as we found positive associations between the total score of the PBA on the one hand, and escape and suicidal ideations, parental neglect, and parental violence on the other hand. We perfectly replicated the medium to large coefficients previously found in the study of Mikolajczak et al. (2018) conducted with French-speaking parents. Alongside the psychometric suitability of the PBA, which has been supported in the current research, our findings again shed light on the detrimental impact of parental burnout on the parents themselves (Manrique-Millones, 2022) and their children. The current results suggest that escape and suicidal ideations, parental neglect, and parental violence are three specific consequences of parental burnout, not only in Western countries, but potentially universally.

For parents, escape or suicidal ideations are triggered by extremely stressful situations in which the person can feel humiliated, ashamed, and unsuccessful (Navarro-Gómez, 2017). In the specific case of parental burnout, parents may have suicidal thoughts in a desperate attempt to escape from such emotional reactions and flee from their strenuous and increasingly demanding role as parents. Other detrimental consequences of parental burnout concern children. When parental burnout strikes, parents, in their eagerness to escape the situation, reduce their levels of involvement to the point of abandoning their responsibilities. This has been documented in investigations within the burnout field, in which excessive demands cause loss of effectiveness leading to neglectful behavior within the work environment (Montero-Marín et al., 2013). Therefore, it would not be surprising for parents with a high level of exhaustion, and lack of empathy, to ignore their children’s needs (Mikolajczak et al., 2019).

Lastly, we corroborated a rather concerning association between parental burnout and parental violence. This may occur because exhausted parents become emotionally distant from their children (Blanchard et al., 2020), a situation that facilitates the use of violence as part of their conflict handling. This is of particular importance in contexts where violent parenting behavior is prevalent as a problem-solving approach. In the words of the Regional Director of UNICEF in Latin America and the Caribbean: “*In Latin America 2 of every 3 children under 5 are victims of different forms of violence in their home*” (Bergua et al., 2018). It should be noted that most of the countries in Latin America have specific legislations that ban physical punishment in different settings such as the home, school, or daycare centers. But while a comprehensive legislation is fundamental, it continues to be insufficient to extirpate violence against children (UNICEF-América Latina y El Caribe, 2018).

Consequently, it is critical to deliver a prevention and an intervention plan dedicated to exhausted parents. Prevention of parental burnout begins with awareness of the phenomenon. This means informing parents and professionals about the nature of the symptoms, their prevalence, their history, and their consequences for the parent, the partner, and the children. It is essential to break the taboo around parental burnout because the symptoms cause shame and guilt in suffering parents. They do not feel authorized to seek help from their surroundings or from a professional. Therefore, information and awareness campaigns should be organized in Latin America, where parental burnout is still an unknown subject for parents and professionals. Beyond the prevention of parental burnout, intervention programs should also be proposed in Latin America. A randomized controlled trial has been published showing that the symptoms of parental burnout and its consequences in terms of neglect and violence can be significantly reduced (Brianda et al., 2020). Two different approaches have been tested to treat parental burnout: a directive approach aimed at reducing stressors and increasing parents’ resources, and a non-directive approach based on active listening and empathy giving parents a space for unconditional acceptance.

### Limitations and Perspectives

The results of the current research show that the PBA evidenced very good psychometric properties across Spanish-speaking countries, and it is therefore valid and desirable for national and cross-cultural research. Nevertheless, these findings should be assessed in light of the following limitations. First, we reported that the samples of Ecuador, Argentina, and Uruguay had to be removed from the ESEM analyses. The bifactor ESEM framework is rather complex as these analyses included much more parameters than CFAs. As a result, ESEM analyses are less suitable with small or not-that-well-defined samples where model non-convergence is more likely to arise (Marsh et al., 2010). We recommend supplementing the approach implemented here with other approaches, including qualitative methods that would allow to study the phenomenon in smaller samples and to explore in depth the culture-specific aspects of the construct.

Second, although we have suggested that culture may play a role in the mean level of parental burnout observed in each country, it is important to be cautious in interpreting these differences. Further research is necessary to make a comparison with other countries not included in this cross-cultural research (in Asia, Africa, and Europe), and to use additional cultural measures. Third, although demographic characteristics were similar among countries, women were overrepresented. This may be a side effect of differing levels of parental involvement between mothers and fathers, although this could be a consequence of a change in gender roles, as presented in the literature (e.g., Olavarría, 2003). Similarly, the parents’ age and number of children were not controlled for. Although we cannot completely rule out the possibility that these factors influenced the prevalence of parental burnout in the current study, sociodemographic factors explained the low amount of variance in parental burnout in previous research (e.g., Mikolajczak et al., 2017; Sorkkila and Aunola, 2020) as well as in cross-cultural research (Roskam et al., 2021, 2022). Fourth, participants mainly came from urban zones of each country’s capital. These samples cannot be considered representative of the entire population in each country either, which could limit the generalizability of the findings. However, there is now strong empirical evidence that sociodemographic characteristics explain a very small amount of variance in parental burnout (Mikolajczak et al., 2017; Arikan et al., 2020; Matias et al., 2020; Mousavi et al., 2020; Sodi et al., 2020; Szczygieł et al., 2020; Roskam et al., 2021). Chances are that the differences discovered across countries were not due to sociodemographic correlates.

Fifth, because of the small sample size in some countries of fathers, and to a lesser extent, of mothers, we were unable to test measurement invariance in each of the five countries separately. Instead, measurement invariance was tested on the pooled sample. Larger samples of fathers would be desirable in future research. Sixth, the use of self-reported measures is a limitation even if parental burnout or suicidal thoughts are subjective conditions requiring such types of instruments. Nevertheless, the measure of parental neglect and violence may be biased by social desirability. Although we did not control for social desirability in Study 2, we can rely on previous findings where the consequences of parental burnout in terms of suicidal ideations, parental neglect, and parental violence were studied (e.g., Mikolajczak et al., 2019). The authors controlled for social desirability and showed that their conclusions held when including social desirability in their three-wave longitudinal model. As in this study, it is likely that the confidential and anonymous nature of our research contributed to providing valid data (Brener et al., 2003). Sixth, depending on the financial or human resources, time frame, and local constraints of each country, the recruitment method varied (i.e., paper-and-pencil or online version, or a combination of both). Although this may be a bias, previous studies reported that measurement equivalence holds across different modes of data collection in the domain of burnout (Campos et al., 2011), parenting behavior (Manrique Millones et al., 2014), or large samples in other disciplines (Revilla, 2013). Finally, while the current results provide solid evidence of the predictive validity of the Spanish version of the PBA, research on the outcomes of parental burnout in Spanish-speaking countries must be expanded. In addition to the potential universality of escape and suicidal ideations, parental neglect, and parental violence as consequences of parental burnout, it is possible that parental burnout predicts other culture-specific consequences in different countries.

## Conclusion

Previous studies have demonstrated the good psychometric properties of the PBA as an instrument for measuring parental burnout in different cultural contexts. These studies have been based on classical confirmatory factor analysis. In this manuscript, we used the bifactor ESEM approach, which provides complementary evidence. In Study 1, we obtained good fit indices supporting a general factor both in the pooled sample and in each country independently. In addition, measurement invariance held across the five countries and gender, and the reliability coefficients were high. In Study 2, we documented the predictive validity of the PBA by reporting moderate to large associations between parental burnout and suicidal and escape ideations, parental neglect, and parental violence toward the offspring. Although we have noted the limitations of our research, the psychometric properties of the PBA in Spanish-speaking countries are strongly supported. The influence of culture on parental burnout is a fascinating research perspective that needs further development. And the instrument validated here provides a solid foundation for such investigations.

## Data Availability Statement

The raw data supporting the conclusions of this article will be made available by the authors, without undue reservation.

## Ethics Statement

An institutional review board provided ethical clearance for the research in each country [i.e., Spain (Bioethics Committee from The National Distance Education University); Perú (The Research Committee of the San Martín de Porres University); Chile (Ethics Committee from the Autonomous University of Chile, 71-18); Uruguay (Ethics Committee of the Faculty of Psychology of the University of the Republic); Ecuador (Institutional Review Commission of the Catholic University of Ecuador); Cuba (Research Ethics Committee of the Faculty of Psychology of the University of Havana, Dossier N-3); and Costa Rica (Scientific Ethics Committee of the University of Costa Rica, N93 VI-1071-2018)]. Note that in Argentina, no ethical clearance was required for the research in accordance with the local legislation and institutional requirements. The patients/participants provided their written informed consent to participate in this study.

## Author Contributions

DM-M was the lead author, coordinated the Spanish-speaking countries, conceptualized and developed the study, wrote a first draft of the manuscript, and subsequently contributed to the rewriting and refinements. IR and MM conceptualized the study, coordinated the IIPB consortium and the data collection, merged the datasets, gave critical insight revising the manuscript, and provided funding acquisition. DM-M, GV, and SD-L conducted data analysis. DM-M, RM-R, RR, MAb, DO, ME, PP-D, MS, CP-M, JT, MAr, MV, MIM, RS, CM-B, AP-S, FS-Q, and PS were responsible for the data acquisition and made a substantial intellectual contribution to the work. All authors have approved the final version of the manuscript and agreed to be accountable for all aspects of their work.

## Conflict of Interest

The authors declare that the research was conducted in the absence of any commercial or financial relationships that could be construed as a potential conflict of interest.

## Publisher’s Note

All claims expressed in this article are solely those of the authors and do not necessarily represent those of their affiliated organizations, or those of the publisher, the editors and the reviewers. Any product that may be evaluated in this article, or claim that may be made by its manufacturer, is not guaranteed or endorsed by the publisher.
