# Supplementary material for: Parental Burnout Assessment (PBA) in Different Hispanic Countries: An Exploratory Structural Equation Modeling Approach
Source: Front Psychol. 2022 Apr 7;13:827014. doi: 10.3389/fpsyg.2022.827014 (PMC9022033; doi:10.3389/fpsyg.2022.827014)
Supplement: Supplementary file 1 [file Table_1.DOCX]

**Parental Burnout Assessment**

**(PBA)**

Los niños son una fuente importante de satisfacción y alegría para sus padres. Al mismo tiempo, también pueden ser una fuente de agotamiento para algunos (y esto no es contradictorio: la satisfacción y el agotamiento pueden coexistir, uno puede amar a los hijos y, sin embargo, sentirse agotado en su rol como padre/madre). El siguiente cuestionario es sobre el agotamiento que se puede sentir como padre/madre. Elija la respuesta que mejor se adapte a sus sentimientos personales. No hay una respuesta buena o mala. Si nunca ha tenido este sentimiento, elija "Nunca". Si ha tenido este sentimiento, indique con qué frecuencia lo siente eligiendo la respuesta que mejor se ajuste. Marque con una X el casillero según su opinión en cada caso.

|  | Nunca | Algunas veces al año o menos | Una vez al mes o menos. | Algunas veces por mes | Una vez por semana | Algunas veces a la semana | Cada día |
| --- | --- | --- | --- | --- | --- | --- | --- |
| 1. Estoy tan cansado(a) por mi rol de padre/madre que tengo la impresión que dormir no es suficiente para recuperarme. |  |  |  |  |  |  |  |
| 1. Siento que ya no me reconozco como padre/madre. |  |  |  |  |  |  |  |
| 1. Siento que estoy al límite de mis fuerzas a causa de mi rol de padre/madre. |  |  |  |  |  |  |  |
| 1. No tengo energía para cuidar a mi(s) hijo(s). |  |  |  |  |  |  |  |
| 1. Siento que ya no soy el buen padre/madre que solía ser para mi (s) hijo (s). |  |  |  |  |  |  |  |
| 1. No puedo soportar más mi rol de padre/madre. |  |  |  |  |  |  |  |
| 1. Tengo la sensación de estar sobrecargado como padre/madre. |  |  |  |  |  |  |  |
| 1. Tengo la impresión de que estoy cuidando a mi(s) hijo(s) en piloto automático. |  |  |  |  |  |  |  |
| 1. Siento que realmente no puedo más como padre/madre. |  |  |  |  |  |  |  |
| 1. Cuando me levanto por la mañana y tengo que enfrentar otro día con mi(s) hijo(s), me siento de antemano agotado(a). |  |  |  |  |  |  |  |
| 1. No siento placer al estar con mi(s) hijo(s). |  |  |  |  |  |  |  |
| 1. Me siento saturado(a) como padre/madre. |  |  |  |  |  |  |  |
| 1. Me digo a mí mismo que ya no soy el padre/la madre que solía ser. |  |  |  |  |  |  |  |
| 1. Solo hago justo lo que hace falta para mi(s) hijo(s), pero nada más. |  |  |  |  |  |  |  |
| 1. Mi rol de padre/madre agota todos mis recursos. |  |  |  |  |  |  |  |
| 1. Ya no soporto más ser padre/madre. |  |  |  |  |  |  |  |
| 1. Estoy avergonzado(a) del padre/madre en el que me he convertido. |  |  |  |  |  |  |  |
| 1. Ya no estoy orgulloso(a) de mi como padre/madre. |  |  |  |  |  |  |  |
| 1. Tengo la impresión de que ya no soy el/la mismo(a) cuando me relaciono con mi(s) hijo(s). |  |  |  |  |  |  |  |
| 1. Ya no logro mostrar a mi(s) hijo(s) cuanto los amo. |  |  |  |  |  |  |  |
| 1. Me agota pensar en todo lo que tengo que hacer para mi(s) hijo(s). |  |  |  |  |  |  |  |
| 1. Tengo la impresión de que, fuera de las rutinas (desplazamientos, hora de acostarse, comidas), no logro involucrarme más con mi(s) hijo(s). |  |  |  |  |  |  |  |
| 1. Estoy en modo de supervivencia en mi rol como padre/madre. |  |  |  |  |  |  |  |
